# Zanubrutinib in patients with treatment‐naïve or relapsed/refractory Waldenström macroglobulinemia: An expanded‐access study of 50 patients in the United States

**DOI:** 10.1002/jha2.619

**Published:** 2023-01-11

**Authors:** Jorge J Castillo, Edwin C Kingsley, Mohit Narang, Habte A Yimer, Constantin A Dasanu, Jason M Melear, Morton Coleman, Charles M Farber, Jonah Shulman, Emily H Mantovani, Xiaowei Zhang, Aileen Cohen, Jane Huang

**Affiliations:** ^1^ Dana‐Farber Cancer Institute Boston Massachusetts USA; ^2^ Comprehensive CancerCenters of Nevada Las Vegas Nevada USA; ^3^ US Oncology Research Maryland Hematology Oncology Columbia Maryland USA; ^4^ Texas Oncology US Oncology Research Tyler Texas USA; ^5^ Lucy Curci Cancer Center Eisenhower Health Rancho Mirage California USA; ^6^ US Oncology Research Texas Oncology, Austin Midtown Austin Texas USA; ^7^ Clinical Research Alliance Weill Cornell Medicine New York New York USA; ^8^ Atlantic Hematology Oncology Morristown Medical Center Morristown New Jersey USA; ^9^ Icahn School of Medicine at Mount Sinai New York New York USA; ^10^ BeiGene (Beijing) Co. Ltd. Beijing China and BeiGene Inc San Mateo California USA

**Keywords:** BTK inhibitor, Waldenström macroglobulinemia, zanubrutinib

Bruton tyrosine kinase inhibitors (BTKi) are effective treatments for patients with Waldenström macroglobulinemia (WM) [1]. In the ASPEN study (NCT03053440), zanubrutinib, a next‐generation BTKi [2, 3], demonstrated fewer toxicities and comparable efficacy versus the first‐generation BTKi, ibrutinib, in patients with WM [1]. Zanubrutinib is approved for the treatment of WM at doses of 160 mg twice daily (BID) or 320 mg once daily (QD) in many countries, including the United States (US) and Europe [4, 5].

This phase 2 expanded‐access study (NCT04052854) evaluated zanubrutinib 320 mg daily (160 mg BID or 320 mg QD per investigator selection) in patients with WM who had relapsed/refractory (R/R) disease or were treatment naïve (TN) and considered unsuitable for standard chemotherapy. Treatment continued in 28‐day cycles until progressive disease (PD), unacceptable toxicity, death, study termination by the sponsor, or commercial availability of zanubrutinib, among other reasons. The study was conducted per the Declaration of Helsinki and the International Conference on Harmonisation Guidelines for Good Clinical Practice and approved by institutional review boards. Informed consent was obtained from all participants.

The primary objective was to provide real‐world experience with zanubrutinib in patients with WM. Secondary objectives included limited assessment of safety and efficacy. Select adverse events (AEs) collected included all serious and grade 3/4 AEs and key AEs at any severity level: anaemia, atrial fibrillation/flutter, haemorrhage, hypertension, infections, major haemorrhage, myalgias, arthralgias, neutropenia, second primary malignancies, thrombocytopenia, and tumour lysis syndrome. Efficacy reporting per investigator was limited to at least every 6 months per modified Owen criteria.[6] The sponsor terminated the study in July 2021 upon commercial availability of zanubrutinib, and on‐treatment patients could continue zanubrutinib through a patient‐assistance programme.

Seventeen patients with TN WM and 33 patients with R/R WM were enrolled at 10 US academic and community medical centres. Overall, 54% of patients were men, the median age was 72 years (range, 47–93), and 82% were aged > 65 years. Most patients had intermediate (54%) or high‐risk (40%) diseases. Patients with R/R WM had a median of two prior treatments (range, 1–5) and began zanubrutinib at a median of 7.7 years (range, 0.7–25.2) after initial diagnosis. Forty‐one patients were dosed at 160 mg BID and nine at 320 mg QD. The median treatment duration was 9.2 months (range, 1.4–20.0), with a median follow‐up of 12.9 months (range, 1.8–20.0).

Forty‐one patients were efficacy evaluable; of these, 39% (95% CI, 24.2–55.5) achieved a best overall response (BOR) of very good partial response (VGPR). Response rates were similar in patients with TN or R/R disease and between the two dosing regimens (Table 1). Nine patients were excluded from the efficacy evaluable population. These patients discontinued before reaching the first protocol‐specified response assessment (eight were on study < 6 months at study termination and transitioned to commercial zanubrutinib). Median progression‐free survival and overall survival were not met owing to the short follow‐up.

**TABLE 1:** Best overall response by investigator assessment in efficacy evaluable population

	**Patients**		**Dose**		
**Best overall assessment, *n* (%)**	**TN (*n* = 11)**	**R/R (*n* = 30)**	**160 mg BID (*n* = 33)**	**320 mg QD (*n* = 8)**	**Overall** * **(*N* = 41)**
Very good partial response	3 (27)	13 (43)	13 (39)	3 (38)	16 (39)
Partial response	4 (36)	10 (33)	12 (36)	2 (25)	14 (34)
Minor response	1 (9)	4 (13)	4 (12)	1 (13)	5 (12)
Stable disease	2 (18)	0 (0)	1 (3)	1 (13)	2 (5)
Progressive disease	1 (9)	3 (10)	3 (9)	1 (13)	4 (10)
Very good partial response or complete response	3 (27)	13 (43)	13 (39)	3 (38)	16 (39)
Major response rate^†^	7 (64)	23 (77)	25 (76)	5 (63)	30 (73)
Overall response rate^‡^	8 (73)	27 (90)	29 (88)	6 (75)	35 (85)

* Nine patients were excluded from the efficacy evaluable population, as they discontinued prior to the first response assessment without clinical PD or death; 41 patients, who had ≥1 response evaluation while in the study, were included in the efficacy evaluable population.

^†^ Partial response or better.

^‡^ Very good partial response, partial or minor response.

Abbreviations: BID, twice daily; PD, progressive disease; QD, once daily; R/R, relapsed/refractory; TN, treatment naïve.

Thirty‐eight (76%) patients experienced ≥1 treatment‐emergent AE (TEAE); 13 (26%) experienced grade ≥3 TEAE, and 36 (72%) experienced ≥1 TEAE of special interest (Table 2). No new safety signals were observed, and the safety profile was similar between the two dosing regimens. Most (76%) patients transitioned to commercial zanubrutinib after study termination. TEAEs were generally manageable with standard therapy. Three patients discontinued zanubrutinib owing to protocol‐specified AEs including grade 1 skin haemorrhage, grade 4 pericardial effusion/grade 3 pleural effusion, and grade 3 soft tissue sarcoma. Four patients had a dose reduction owing to protocol‐specified AEs including grade 3 pruritis, grade 3 fatigue, grade 1 contusion/skin haemorrhage, and grade 2 arthralgia (Table 2). Diarrhoea was not assessed per protocol, but one patient had grade 1 diarrhoea leading to dose reduction, and another experienced grade 2 diarrhoea and grade 3 fatigue, leading to dose reduction and discontinuation. No dose modifications resulted from coronavirus disease 2019.

**TABLE 2:** Adverse events of special interest and dose modifications due to adverse event

**Adverse events, *n* (%) (safety population)**	**Zanubrutinib 160 mg BID (*n* = 41)**	**Zanubrutinib 320 mg QD (*n* = 9)**	**Overall (*N* = 50)**
≥1 TEAE of special interest* (category)	31 (76)	5 (56)	36 (72)
Anaemia	2 (5)	0 (0)	2 (4)
Atrial fibrillation and flutter	1 (2)	0 (0)	1 (2)
Haemorrhage	16 (39)	3 (33)	19 (38)
Hypertension	5 (12)	0 (0)	5 (10)
Infections	15 (37)	2 (22)	17 (34)
Neutropenia	2 (5)	0 (0)	2 (4)
Second primary malignancies	4 (10)	0 (0)	4 (8)
Thrombocytopenia	1 (2)	1 (11)	2 (4)
Tumour lysis syndrome	0 (0)	0 (0)	0 (0)
Grade ≥3 TEAEs of special interest	7 (17)	1 (11)	8 (16)
Hypertension	4 (10)	0 (0)	4(8)
Hypertension	4 (10)	0 (0)	4 (8)
Procedural hypertension	1 (2)	0 (0)	1 (2)
Infection	3 (7)	1 (11)	4 (8)
Pneumonia	1 (2)	1 (11)	2 (4)
COVID‐19 pneumonia	1 (2)	0 (0)	1 (2)
Cellulitis	1 (2)	0 (0)	1 (2)
*Staphylococcal bacteraemia*	1 (2)	0 (0)	1 (2)
Atrial fibrillation and flutter	1 (2)	0 (0)	1 (2)
Neutropenia	1 (2)	0 (0)	1 (2)
Second primary malignancy	1 (2)	0 (0)	1 (2)
Soft tissue sarcoma	1 (2)	0 (0)	1 (2)
TEAEs leading to treatment discontinuation†	1 (2)	2 (22)	3 (6)
TEAEs leading to dose reduction	3 (7)	1 (11)	4 (8)

^*^TEAE of special interest categories were defined as haemorrhage, atrial fibrillation/flutter, hypertension, second primary malignancies, tumour lysis syndrome, infections, neutropenia, thrombocytopenia, and anaemia. Patients with multiple events for a given category are counted only once for each category.

^†^One patient had R/R WM and was diagnosed with treatment‐related serious grade 3 soft tissue sarcoma on study day 330; the event was treated with chemotherapy, and after 55 days the adverse event progressed to grade 5, leading to death. Another had TN WM and developed treatment‐related serious grade 4 pericardial effusion and grade 3 pleural effusion on study day 227, and treatment was discontinued owing to the events on study day 231. The events lasted for 13 days and were resolved by concomitant procedure and medication. The third patient had R/R WM and developed treatment‐related grade 1 skin haemorrhage on study day 50; the dose was immediately interrupted for 7 days, then reduced to 160 mg QD, and ultimately withdrawn on study day 111. The event lasted 65 days and was resolved 3 days after treatment discontinuation.

Abbreviations: BID, twice daily; QD, once daily; R/R, relapsed/refractory; TEAE, treatment‐emergent adverse event; TN, treatment naïve; WM, Waldenström macroglobulinemia.

Two patients with R/R disease died > 30 days after their last dose owing to indeterminate cause and AE (soft tissue sarcoma). The patient with sarcoma discontinued zanubrutinib after 11 months to receive sarcoma treatment including ifosfamide, doxorubicin and mesna and died of sarcoma 55 days after zanubrutinib discontinuation. The investigator assessed the event as possibly related to zanubrutinib.

Patients’ characteristics were similar to those of the ASPEN population[1]; however, key differences included age distribution, Eastern Cooperative Oncology Group performance status (ECOG PS), disease course duration, and prognosis. The current study had a lower proportion of younger patients (18% vs. 40%, ≤65 years), worse baseline ECOG PS (ECOG 0, 14% vs. 45%), less low‐risk disease (4% vs. 17%), and patients with R/R WM were approximately 2.5 years more advanced in their disease course (7.7 vs. 5.3 years).[1]

Response rates were also similar to those reported in ASPEN. VGPR was higher in the efficacy evaluable population compared with the investigator‐assessed response in the ASPEN ITT population (39% vs. 28%), with comparable major response rates (73% vs. 77%). The overall response rate (ORR) was lower in the current study (85% vs. 95%).[1] Overall, four (10%) patients had a BOR of PD, which was higher than the PD rate (1%) observed in ASPEN[1] and accounted for the lower ORR observed. This difference can be attributed to response assessments being reported monthly for the first year in ASPEN, compared to being conducted per local clinical practice but only reported per protocol every 6 months in the present study; therefore, any responses that may have been achieved between required response assessments (e.g., months 1–5 or months 7–11) would not have been captured. In the four patients with a BOR of PD, three had IgM levels during the first six cycles that indicated a response before the first assessment; the fourth patient started zanubrutinib 17 years after the initial WM diagnosis and experienced PD after cycle one.

Study limitations included a small sample size, unblinded design, short follow‐up duration, infrequent response assessments, unreported gene sequence status, and US‐only enrolment. Although this is a non‐comparative study, the results of zanubrutinib in patients with R/R or TN WM at 160 mg BID or 320 mg QD are consistent with the previous observations from earlier studies.

## AUTHOR CONTRIBUTIONS

Aileen Cohen and Jane Huang were responsible for the conception and design of the study. Emily H. Mantovani was responsible for supporting study oversight, clinical data review, data compilation and interpretation. Xiaowei Zhang was responsible for data analyses. Jorge J. Castillo, Edwin C. Kingsley, Mohit Narang, Habte A. Yimer, Constantin A. Dasanu, Jason M. Melear, Morton Coleman, Charles M. Farber, and Jonah Shulman recruited patients for the study. All authors drafted and critically reviewed the manuscript. All authors provided final approval of the manuscript.

## CONFLICT OF INTEREST

Jorge J. Castillo reports consulting or advisory roles with AbbVie/Pharmacyclics, BeiGene, Cellectar, Janssen, and Roche/Genentech; and research funding from AbbVie, AstraZeneca, Cellectar, LOXO, Pharmacyclics, and TG Therapeutics, outside the submitted work. Mohit Narang reports consulting or advisory roles with BeiGene, Bristol Myers Squibb, Celgene, DSI, and Takeda; and speakers bureau with BeiGene, Bristol Myers Squibb, and Takeda, outside the submitted work. Habte A. Yimer reports stock/stock options from Karyopharm, and speakers bureau with Amgen, AstraZeneca, BeiGene, GSK, Janssen, Karyopharm, Pharmacyclics, and Sanofi, outside the submitted work. Jason M. Melear reports consulting or advisory roles with TG Therapeutics, and speakers bureau with AstraZeneca and BeiGene, outside the submitted work. Morton Coleman reports stocks/stock options from Immunomedics, and receives research funding from AbbVie, BeiGene, Bristol Myers Squibb, Celgene, Genentech, Gilead, InnoCare, Merck, Pfizer, and Roche, outside the submitted work. Charles M. Farber reports stocks/stock options from Alexion; receiving honoraria from Bristol Myers Squibb; consulting or advisory roles with ADC Therapeutics, BeiGene, Gilead, MorphoSys/Incyte, and TG Therapeutics; speakers bureau with ADC Therapeutics, Genentech, Gilead, MorphoSys/Incyte, Seagen, and TG Therapeutics, outside the submitted work. Jonah Shulman reports receiving honoraria from Seagen and TG Therapeutics, and consulting or advisory roles with Seagen and TG Therapeutics, outside the submitted work. Emily H. Mantovani is an employee of BeiGene, with regard to the submitted work, reports former employment with Bristol Myers Squibb, and reports stocks/stock options from BeiGene and Bristol Myers Squibb, outside the submitted work. Xiaowei Zhang and Aileen Cohen are employees of BeiGene, with regards to the submitted work, and report stocks/stock options from BeiGene, outside the submitted work. Jane Huang was an employee of BeiGene at the time of the study, with regards to the submitted work; reports receiving royalties from BeiGene; receiving support for attending meetings and/or travel from BeiGene and Protara; receiving research funding from BeiGene; patents, royalties, or other intellectual property with BeiGene; a leadership role with BeiGene and Protara; and stock/stock options from BeiGene and Roche, outside the submitted work. Edwin C. Kingsley and Constantin A. Dasanu have no relevant disclosures.
